# Bioenergy crop induced changes in soil properties: A case study on Miscanthus fields in the Upper Rhine Region

**DOI:** 10.1371/journal.pone.0200901

**Published:** 2018-07-26

**Authors:** Yaxian Hu, Gerhard Schäfer, Joëlle Duplay, Nikolaus J. Kuhn

**Affiliations:** 1 State Key Laboratory of Soil Erosion and Dryland Farming on the Loess Plateau, Institute of Soil and Water Conservation, Northwest A&F University, Yangling, China; 2 Physical Geography and Environmental Change, Department of Environmental Sciences, University of Basel, Basel, Switzerland; 3 Université de Strasbourg, CNRS, Laboratoire d'Hydrologie et de Géochimie de Strasbourg UMR 7517, Strasbourg, France; University of Huddersfield, UNITED KINGDOM

## Abstract

Biomass as a renewable energy source has become increasingly prevalent in Europe to comply with greenhouse gas emission targets. As one of the most efficient perennial bioenergy crops, there is great potential in the Upper Rhine Region to explore biomass utilization of Miscanthus to confront climate change and land use demand in the future. Yet, the impacts of Miscanthus cultivation on soil quality have not been adequately explored. This study investigated the soil profiles of five- and 20-year-old Miscanthus fields (1 m depth) as well as grassland for reference in eastern France and Switzerland. The soil organic carbon (SOC) concentrations and δ^13^C compositions of four soil layers (0–10 cm, 10–40 cm, 40–70 cm and 70–100 cm) were determined. The CO_2_ emission rates of the topsoil were monitored for 42 days. Our results showed that Miscanthus, in general, could increase the SOC stocks compared to grassland, but the benefits of SOC sequestration were constrained to the surface soil. Isotopically, the Miscanthus-derived SOC ranged from 69% in the top 10 cm of soil down to only 7% in the 70 cm to 100 cm layer. This result raises the risk of overestimating the total net benefits of Miscanthus cultivation, when simply using the greater SOC stocks near the surface soil to represent the SOC-depleted deep soil layers. The Miscanthus fields had greater CO_2_ emissions, implying that the Miscanthus fields generated greater ecosystem respiration, rather than larger net ecosystem exchanges. Compared to the grassland soils, the surface soils of the Miscanthus fields tended to have a risk of acidification while having higher concentrations of phosphorus and potassium, calling for the inclusion of soil characteristics and SOC stability when evaluating the impacts of long-term Miscanthus cultivation on both current and future land use changes.

## Introduction

Carbon sequestration and fossil fuel offset by bioenergy crops are an important component to reducing Greenhouse Gas (GHG) emissions [1]. As a renewable energy source, bioenergy crops have become increasingly prevalent in Europe to ensure a sustainable energy supply [2–4]. However, Lal [5] noted that there is no such thing as a free biofuel from crop residues, and biofuel feedstock must be obtained by establishing site-specific plantations. Miscanthus is one of the most efficient perennial bioenergy crops, due to its great adaptability to different environments, long life time, high yield, low fertilizer and pesticide requirement, and greater photosynthetic efficiency [6–8]. Achieving the sustainable utilization of regional biomass potential requires a full understanding of the impacts of Miscanthus cultivation, not only on the economy but also on the environment.

Substantial research has been devoted to investigating the conversion pathways, side products control, air pollutant emissions, and economic benefits from Miscanthus [9–11]. In particular, from the environmental point of view, benefits with respect to high water use efficiency, little requirement of nutrients, and year round cover to reduce soil erosion risk, have made Miscanthus a preferred bioenergy crop [6,8,12]. Nevertheless, GHG emissions during land use changes might render the net environmental benefits less favorable, especially when a large-scale conversion occurs [13,14]. Such negative environmental impacts could be further impacted by GHG emissions from applying nitrogen fertilizer, fossil energy consumption during tillage operation, production, storage, transportation and pelletizing [9,10,15,16].

The actual benefit of Miscanthus, in addition to reducing emissions by burning biomass instead of fossil fuels, is the increase of soil SOC. With the removal of the aboveground biomass for energy generation, abundant residues accumulate in the surface soil. Vigorous development of deep roots is also a major input of C entering the SOC pool. Due to the absence of tillage practices for vertical exchanges across soil layers, long-term Miscanthus cultivation is very likely to have vertical patterns in changes of the soil structure and mineral composition. Consequently, these changes would change the soil nutrient status and sustainability over the soil depth [17–21]. In terms of SOC stocks, most of the research claimed positive benefits such as mitigating climate change with additional atmospheric CO_2_ sequestration [22–24]. However, Hansen et al. [25,26] noted that Miscanthus-derived SOC mainly consisted of particulate organic matter, which was not very stable with regards to sequestering atmospheric CO_2_ into the soil. The labile quality of newly sequestered SOC may thus bear unknown uncertainties in terms of overall GHG emissions [21,26]. However, Foereid et al. [27] argued that the Miscanthus-derived organic matter was at least as stable as grassland-derived organic matter and that the turnover time of the organic matter increased with time under Miscanthus cultivation. These discrepancies urge a close investigation of the stability and potential mineralization of Miscanthus-derived SOC.

In the Upper Rhine Region shared by France, Switzerland and Germany, 37% of the total area is arable land used for agriculture. To offset fossil fuels, there is an increasing interest in growing different types of bioenergy crops. Accordingly, Miscanthus trials have been encouraged and increased in the Upper Rhine Region since the 1990s [28]. In this study, soil profiles from three Miscanthus sites of different ages in the Upper Rhine Region were investigated. By comparing the vertical distribution of SOC, δ^13^C compositions and CO_2_ emissions across soil depths in Miscanthus fields with those in grasslands, this study aimed to: 1) detect the fractions of Miscanthus-derived SOC in the different soil layers and 2) evaluate the changes in soil characteristics such as pH, phosphorous (P) and potassium (K) after long-term Miscanthus cultivation. This information enabled the assessment of Miscanthus on both SOC stocks and net soil C uptake, and other impact on soil and environmental quality.

## Materials and methods

### Study site

Two silty loams with three cultivation durations from two fields were investigated in this study (Table 1). The first field was sampled in May 2014 from the Farm Niedererweiher (47°41’N, 7°10’E, 297 m altitude), near Ammerzwiller, Alsace, France. Miscanthus was planted 5 years ago and 20 years ago; thus, the sites are hereafter referred to as “A-5”, and “A-20”. Undisturbed grassland (hereafter termed as “A-grass”), approximately 50 m away from the 20-year Miscanthus was of the same soil texture and previous vegetation and was sampled as a reference site. The annual rainfall near the Farm Niedererweiher was 773 mm, with approximately 223 mm falling in April, May and June, during which the average maximum temperature was 19.6°C (weather station in Mulhouse, 1981–2010, Metéo France).

**Table 1:** Summary of soil types, names, locations, tillage history and yearly yield of the three treatments on two fields.

Soil type	Farm	Location	History of Miscanthus cultivation(ca.)	Previous plants	Dry yield on average (Mg ha^-1^)
A-5	Niedererweiher	Ammerzwiller, Alsace, France	5 years	Grass	15
A-20	Niedererweiher	Ammerzwiller, Alsace, France	20 years	Grass	15
M-20	Untergruth	Münchenstein, Basel-Land, Switzerland	20 years	Wheat, barley, grass	6

After preliminary analysis of soil samples collected in May, a second silty loam was sampled in November 2014 to verify the applicability of Miscanthus-induced changes in soil properties in other fields of the Upper Rhine Region. The second silty loam was from a field with 20 years of Miscanthus growth on the Farm Untergruth (47°30’N, 7°38’E, 316 m altitude) in Münchenstein, Canton Basel-Land (Switzerland). This site was referred to as “M-20”. A grassland right beside the Miscanthus field was also sampled as a reference site, hereafter referred to as “M-grass”. Prior to Miscanthus cultivation, the field had different land-uses, i.e., rotation of arable (wheat and barley) and grass (Table 1). The annual precipitation near Münchenstein was 842 mm, with approximately 249 mm falling in April, May and June, during which the average maximum temperature was 19.2°C (weather station at Binningen, 1981–2010, Meteo Schweiz).

Every year, approximately 6 Mg·ha^-1^ of dried Miscanthus was harvested from the Münchenstein field, and approximately15 Mg·ha^-1^ of Miscanthus was harvested from the 5-year-old and 20-year-old fields in Alsace (Table 1). For all of the fields, after harvesting in March each year, approximately 30-cm high Miscanthus stubble was left standing. No fertilizer of any type was ever applied to Miscanthus fields or grasslands. For the Farm Niedererweiher in Alsace, 500 kg ha^-1^ agricultural lime was applied every 5 years to neutralize the soil acidity. No agricultural lime was applied to the field at Münchenstein.

### Soil and plant sampling

Soil cores were sampled by a 1-m long soil core sampler on A-20, M-20 and grasslands, and separated into four layers (0–10 cm, 10–40 cm, 40–70 cm and 70–100 cm). Due to the failure of the core sampler on the field, A-5 was only sampled to 30 cm by 0–10 cm, 10–20 cm and 20–30 cm. In addition, loose soil on the surface was observed to be well blended with semi-decomposed residues; therefore, this was collected separately (termed as “surface”). The sampling sites on each field were randomly chosen, and three replicates were taken from each field. Surface soils were also collected by standard-sized cylinders (volume of 95 cm^3^) to determine the soil bulk density. Different parts of Miscanthus plants were also collected at each soil sampling spot. Roots, stems and leaves were separately collected, immediately dried at 40°C and then ground into powder to be ready for the stable isotope measurements.

### Laboratory analysis

All of the soil samples were stored in a cold chamber at 4°C during transport to limit bioactivities. Immediately after sampling, P and K were extracted in CO_2_ saturated water (1:10). All of the concentrations of P and K were measured using an ion chromatography (Metrohm 761 Compact IC with the Auto-sampler 698, Herisau, Switzerland). Due to the limited amount of soil from individual layers, replicated samples were mixed and measured to represent the nutrient status and pH value of each layer. The pH values were determined in a 0.01 M CaCl_2_ suspension (1:2.5) using a SevenExcellence pH metre (Mettler-Toledo International, Columbus, Ohio, USA).

The CO_2_ emission rates of the topsoil (0–10 cm and 10–40 cm) from both Miscanthus fields and grasslands were measured based on the method described in Robertson et al. [29] and Zibilske [30]. Approximately 25 g of moist soils from the top 10 cm and 10–40 cm from all three fields were immediately incubated at 20°C in flasks with a volume of 200 cm^3^ (flasks open). Visible residues or roots were manually removed. Prior to the soil CO_2_ emission measurements, all of the flasks were sealed using rubber stoppers. A one ml of gas was extracted from the headspace of each sealed flask by a syringe both at the beginning and at the end of the one hour sampling period. Differences in CO_2_ concentrations between the one hour period of time were used to calculate the instantaneous CO_2_ emission rate. The CO_2_ emission rate measurements of the A-5, A-20 and A-Grass were repeated at days 1, 2, 3, 7, and 14, and every 7 days after until 42 days, and the CO_2_ emission rate measurements of the M-20 and M-Grass were repeated at days 1, 8, 15, 22 and 30 (measurements aborted after consistent patterns between Miscanthus field and Grassland were observed). The cumulative CO_2_ emission rates were calculated by linearly extrapolating hourly rates to daily rates and then accumulating over day-intervals into 42 days CO_2_ emission amount. While such crude extrapolation cannot be used to draw any quantitative estimation on longer-term CO_2_ emission potentials, it can provide comparative information on the differences between Miscanthus and grasslands. The CO_2_ concentrations were measured using a SRI8610C Gas Chromatograph (California, USA).

Prior to the CO_2_ emission measurements, the soil samples were not dried and then re-wetted to an arbitrary soil moisture, which would often have unknown effects on soil respiration. Soil samples in this study were directly incubated at the natural moisture contents collected from the field. Although the effects of water potential and other factors could not be separated, incubation at field moisture was considered adequate to reflect the Miscanthus-induced differences in SOC decomposition. During the incubation period, the moist soil samples were weighed every 3 days to monitor their soil moisture, the variation of which was constrained within 1% by re-wetting. At the end of the incubation tests, all the wet samples were dried to calculate their actual soil moisture. Details please see the Table 2.

**Table 2:** Soil moisture of the incubated samples from the Miscanthus and grassland.

	Soil moisture of incubated samples (%)				
	A-grass	A-5	A-20	M-grass	M-20
**0–10 cm**	52.04	51.12	52.64	45.72	43.74
	61.23	50.19	79.58	45.71	33.92
	40.79	68.96	72.73	44.30	56.02
**10–20 cm**	47.83	38.47	51.14	28.20	24.23
	38.76	42.49	45.68	34.89	27.22
	29.65	69.68	42.34	30.23	26.27

The rest of the soil samples that were not used for CO_2_ emission measurements were dried at 40°C until a constant weight was reached. Soil SOC concentrations of all layers were measured using a Leco RC612 (St. Joseph, USA) after removing visible roots and residues. The difference of SOC concentration was calculated from the SOC concentration in each layer of Miscanthus fields minus that of the Grassland. The SOC stocks in the upper 10 cm were calculated by multiplying the SOC concentrations with respective soil bulk density of that layer. The changes of SOC stocks in the upper 10 cm were then deduced from the differences of SOC stocks between the reference grassland and the Miscanthus fields with stand age of 5 or 20 years. The yearly increase rate of SOC was then calculated by normalizing the total increase of SOC stocks in the Miscanthus fields over the 5 or 20 years. The C: N ratios were determined by a Leco CN628 (St. Joseph, USA).

Due to the different molecular structures to convert atmospheric CO_2_ to different phosphoglycerate compounds with different C atoms, C_4_ plants such as Miscanthus have δ^13^C values from -17 to -9‰, while C_3_ plants such as grassland have δ^13^C values from -32 to -20‰ [31]. Therefore, the distinct δ^13^C compositions between C_3_ and C_4_ plants can be used to distinguish the source of C compositions (i.e., from Miscanthus or from grass) in this study. The stable isotope composition of δ^13^C of all the soil layers and different Miscanthus plant parts were analyzed using a Costech elemental analyser coupled to a Delta V Plus (Thermo Fisher) isotope ratio mass spectrometer at the University of California, Merced. The stable isotope compositions of δ^13^C of all Miscanthus plant and root samples were determined by isotope ratio mass spectrometry (EA-IRMS) using an INTEGRA2 Instrument (Sercon Ltd., Crewe, UK) at the Department of Environmental Sciences, University of Basel. All of the standards were referenced to the international standard Pee Dee Belemnite. The stable isotopic compositions were expressed in δ notation (‰) as follows: δ^13^C = [(R_sample_—R_standard_)/ R_standard_] × 1000; where R_sample_ is the ratio of the heavy to the light C (^13^C/^12^C) isotopes in a sample; and R_standard_ is the ratio in a standard [32]. The percentage of SOC derived from Miscanthus was calculated following the isotope mass balance equation in Balesdent and Mariotti [31]:

$$f_{M}=\frac{\delta^{13}\times {SOC}_{MS}-\delta^{13}\times {SOC}_{GS}}{\delta^{13}\times {SOC}_{MP}-\delta^{13}\times {SOC}_{GS}}\times 100\%$$ (1)

where, f_M_ represents the percentage of SOC derived from Miscanthus; the subscript *MS* represents Miscanthus soil, *GS* represents grassland soil, and the *MP* represents Miscanthus plant.

### Statistical analysis

While pair-wise comparisons had the advantages to highlight the differences between the Miscanthus and Grassland, mismatches of extreme values from two fields were very likely to introduce over- or under-representation in data interpretation. Therefore, the C: N ratios, P and K content in Miscanthus and Grassland fields were first sorted in ascending order within group before paired up. All the data analysis was carried out by RStudio software.

## Results

### Soil organic carbon

**Fig 1:**
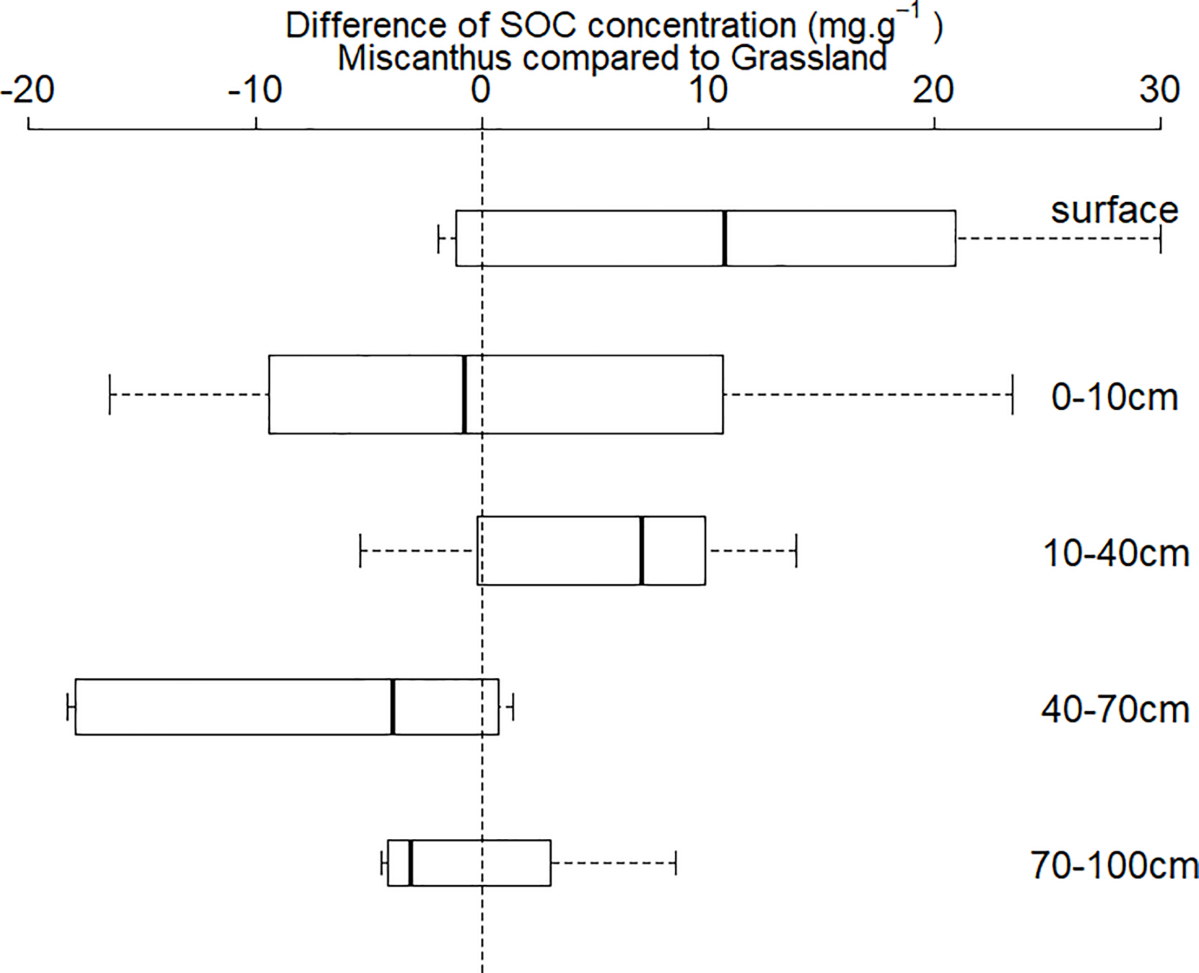
Difference of SOC concentrations in Miscanthus fields compared to the grassland across different soil depths. “Surface” denotes loose soil samples collected on the surface, where semi-decomposed Miscanthus residues were well blended with soil. The SOC of surface samples was measured after removing visible roots and residues.

The SOC concentration from all of the soil layers in the Miscanthus field was compared to that in the grassland (Fig 1, data listed in S1 Table)). For all of the soil layers, the SOC concentrations of Miscanthus samples were generally greater than those of the grassland soils. In addition, the difference of the SOC concentrations decreased with soil depth (Fig 1). When considering only the top 10 cm, the SOC contents in the A-20 and the A-5 were approximately 31.18% and 10.00%, respectively, greater than that on the A-grass. However, such increasing SOC effects were not observed in the Münchenstein field, where a decrease of 13% in SOC content was found on the M-20 (Table 3).

**Table 3:** Comparison of the bulk density, SOC concentrations and SOC stocks in the upper 10 cm on the Miscanthus and Grassland in the three fields. The subscripts indicate the ranges of the values (*n* = 3).

	Bulk density (g cm^-3^)	SOC concentration (mg g^-1^)	SOC stock in upper 10 cm (Mg ha^-1^)	Increase of SOC stock of Miscanthus over Grassland (%)
**A-grass**	0.99_±0.24_	34.38_±4.8_	34.0_±14.2_	-
**A-5**	1.27_±0.09_	29.38_±4.9_	37.4_±9.3_	10.00
**A-20**	1.00_±0.21_	44.54_±5.8_	44.6_±16.4_	31.18
**M-grass**	0.99_±0.10_	29.87_±10.74_	29.6_±14.7_	-
**M-20**	0.96_±0.04_	26.75_±6.01_	25.7_±7.1_	-13.17

### Stable isotope δ^13^C

Given that the variation between different parts of the Miscanthus was minor when compared with the changes of δ^13^C compositions in soil layers, the δ^13^C compositions of Miscanthus roots, leaves and stems were combined as replicates to represent the entire Miscanthus plant. The δ^13^C compositions of all the soil layers on the Miscanthus fields had lower negative values than those of the soils from the grasslands (Table 4, original data listed in S1 Table). Such negative changes were more significant in the upper 10–40 cm and gradually diminished through the soil profile. The fractions of Miscanthus-derived SOC listed in Table 4 showed that the contribution of Miscanthus to current SOC concentrations was up to 69% in the 0–10 cm layer on the A-20 field, while as low as 7.67% in the 70–100 cm layer on the M-20 field.

**Table 4:** The δ^13^C compositions from the Miscanthus soils (MS), the grassland soils (GS), and the Miscanthus plants (MP). The subscripts after “δ^13^C_MS_” and “δ^13^C_GS_” denote the minimum and maximum ranges of the values, and the subscripts after the “δ^13^C_MP_” denote the standard deviation (*n* = 8).

	Layer (cm)	Miscanthus soil δ^13^C_MS_ (‰)	Grass-induced δ^13^C_GS_ (‰)	Miscanthus-induced δ^13^C_MP_ (‰)	Fraction of Miscanthus- derived SOC (%)
**A-5**	**0–10**	-24.46 _±0.05_	-28.04_±0.05_	-12.02_±0.44_	22.37
	**10–20**	-23.70_±0.40_	-26.15_±0.65_	-12.02_±0.44_	17.35
	**20–30**	-25.86_±0.20_	-27.37_±0.85_	-12.39_±0.54_	10.11
**A-20**	**0–10**	-17.09_±2.30_	-28.04_±0.07_	-12.23_±0.35_	69.25
	**10–40**	-22.26_±0.25_	-26.15_±0.68_	-12.23_±0.35_	27.91
	**40–70**	-22.53_±0.25_	-27.37_±0.86_	-12.47_±0.19_	32.49
	**70–100**	-25.02_±0.95_	-26.50_±0.01_	-12.47_±0.19_	10.54
**M-20**	**0–10**	-20.75_±1.15_	-28.55_±0.15_	-12.69_±0.54_	49.19
	**10–40**	-25.07_±0.40_	-27.29_±0.15_	-12.69_±0.54_	15.20
	**40–70**	-24.74_±0.45_	-26.09_±0.20_	-12.55_±0.43_	9.92
	**70–100**	-25.06_±0.15_	-26.10_±0.20_	-12.55_±0.43_	7.67

### C: N ratios, CO_2_ emissions, pH and nutrients

**Fig 2:**
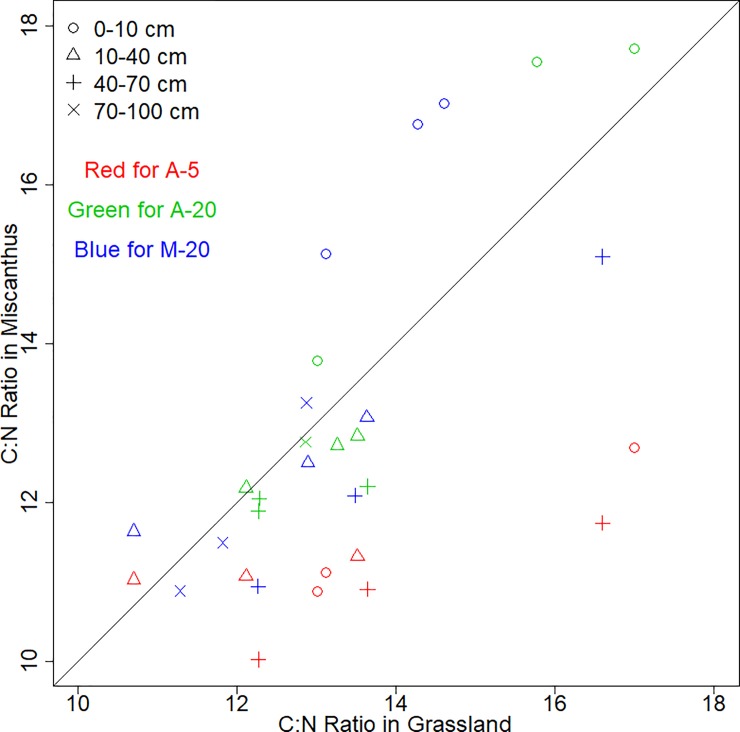
Comparison of the C: N ratios between Miscanthus and grassland soils across all the layers collected from Alsace (A) and Münchenstein (M). The bold line indicates the 1:1 ratio. Note that for the A-5, the red open dots, red open delta and red cross symbols represent the 0–10 cm, 10–20 cm and 20–30 cm.

The C: N ratios of the 20 years old Miscanthus soils were evidently greater than those of the grassland soils in the upper soil layers (Fig 2, data listed in S1 Table). In the lower soil layers, the C: N ratios were either equal or greater in the grassland soils.

**Fig 3:**
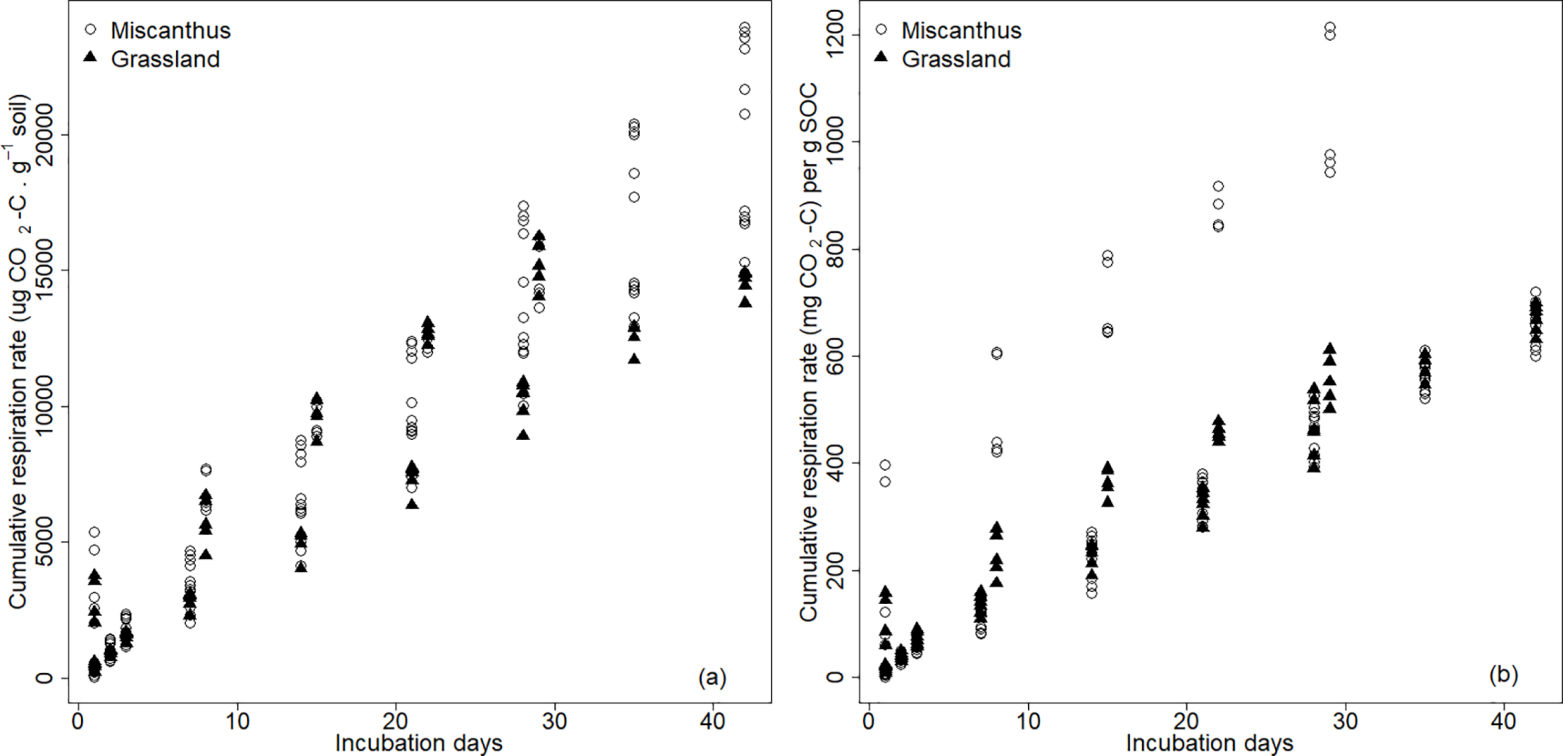
Cumulative CO_2_ emission rates per gram soil (a) and cumulative CO_2_ emission rates per gram SOC (b) measured over the entire incubation periods between grassland and Miscanthus for both the 0–10 cm and 10–40 cm layers.

The CO_2_ emission rates of grassland and Miscanthus soils from both study sites showed instantaneous pulses of CO_2_ emissions during the first three days of measurements, but gradually declined as the incubation proceeded. Their cumulative CO_2_ emissions from both the 0–10 cm and 10–40 cm layers are plotted in Fig 3A (data listed in S1 Table). It shows that the monitored CO_2_ emissions of Miscanthus, in general, were greater than those in grassland in both soil layers. The CO_2_ emission rates per gram SOC were even 73.70% more pronounced in the grassland soil than in the Miscanthus soil (Fig 3B).

**Fig 4:**
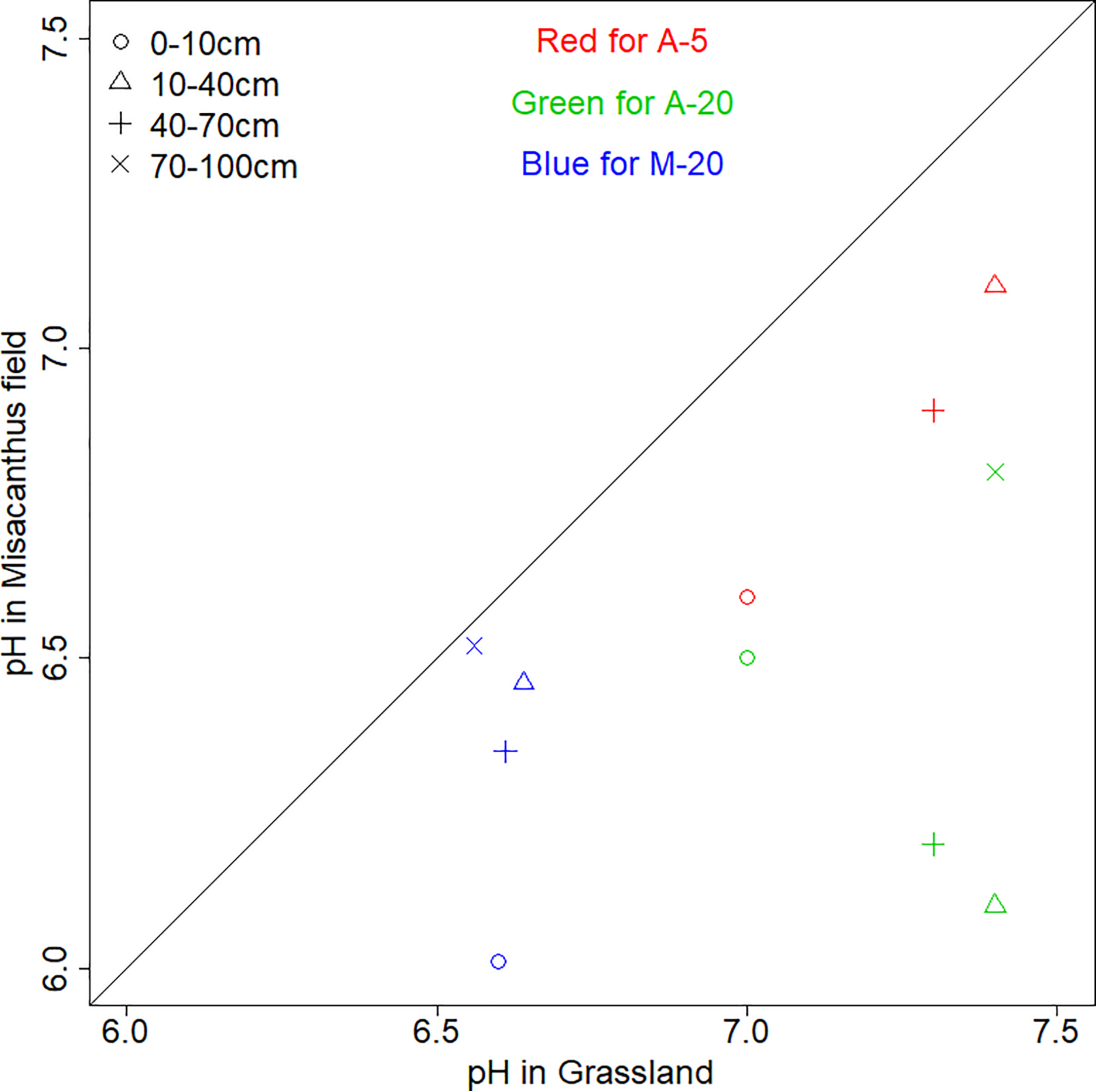
Comparison of pH between Miscanthus and grassland across four different layers collected from all the three fields in Alsace (A) and Münchenstein (M). Bold line indicates the 1:1 ratio. Note that for A-5, red open dots, red open delta and red cross symbols represent 0–10 cm, 10–20 cm and 20–30 cm.

**Fig 5:**
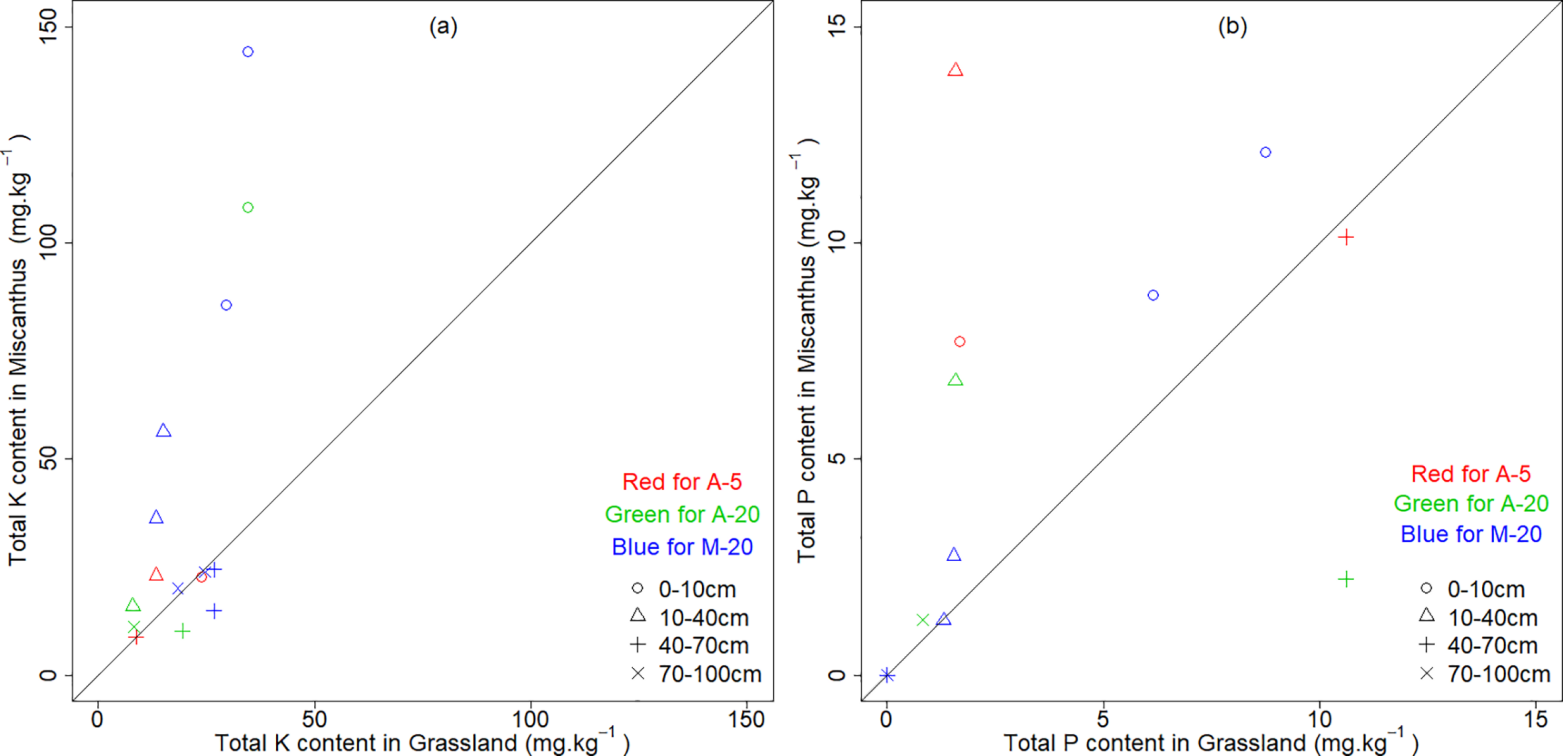
**Pairwise scatterplots of the total K content (a) and total P content (b) between the grassland and the Miscanthus for all the soil layers in Alsace (A) and Münchenstein (M).** Bold lines indicate the 1:1 ratio. Note that for A-5, red open dots, red open delta and red cross symbols represent 0–10 cm, 10–20 cm and 20–30 cm.

The pH values of all four soil depths on both the Miscanthus and grassland fields are illustrated in Fig 4 (data listed in S1 Table). In general, the Miscanthus fields had significantly lower pH values than the grassland fields (*p* < = 0.05, *t*-test). In particular, the pH in the top 10 cm of the Miscanthus field was evidently low. The P and K were also enriched on the surface soil of the Miscanthus fields (Fig 5A and 5B), but only K was significantly greater in the Miscanthus fields (*p* < = 0.05, *t*-test).

## Discussion

### Miscanthus effects on SOC

The decreasing pattern of SOC concentrations (Fig 1) and Miscanthus-derived SOC fractions across soil depths (Table 4) suggested that the C_4_ plant Miscanthus greatly changed the SOC compounds, but that the benefits of Miscanthus for increasing SOC concentration were probably limited to the surface soil. Such a vertically declining pattern was in agreement with the most recent studies [23,25,26,33]. We agreed with their explanation that the greater SOC concentration in the topsoil of the Miscanthus fields was mostly due to the return of residues (loose leaves and approximately 30 cm of stubble in our study), helping to accumulate the SOC near the soil surface. The fractions of Miscanthus-derived SOC observed in this study were, in general, greater than the results reported in Hansen et al. [25]. This was probably caused by the greater potential of the finer-textured silty loams in the Upper Rhine Region to sequester SOC compared to the loamy sand in Hansen et al. [25]. Nevertheless, the increased SOC concentrations (Fig 1) and predominant δ^13^C compositions (Table 4) from the upper soil layers (Fig 3B) cannot be directly translated to the net increase of SOC stocks, especially when considering the declining patterns of SOC concentrations and δ^13^C compositions over soil depths. Therefore, any extrapolation from the changes of topsoil SOC to a net increase of SOC stocks in the whole soil profile should be applied with great caution.

The non-distinguishable CO_2_ emission rates per gram of SOC observed in this study (Fig 3B) indicated a comparable SOC stability in the Miscanthus and grassland soils. While Hansen t al. [25] and Zimmermann et al. [26] suggested that Miscanthus-derived SOC mainly consisted of particulate organic matter, Foereid et al. [27] stated that the Miscanthus-derived organic matter was at least as stable as grassland-derived organic matter. The greater CO_2_ emissions from the Miscanthus soils observed in this study (Fig 3A) were more likely to be a result of the greater SOC concentrations in the upper soil layers (Fig 1). This suggested that the Miscanthus fields had greater uptake and accumulated more atmospheric CO_2_ in the soil than the grassland, thus generating greater ecosystem respiration, rather than larger net ecosystem exchanges.

The slightly slowing down SOC increase rate at the A-20 field as compared to the A-5 field (Table 3) also implied that the benefits of SOC sequestration in surface soil may approach its maximum capacity after 20 years as conceptually proposed in Lal [34]. However, even though the M-20 field was previously under arable rotation, which supposedly had larger potential to sequester SOC, its accumulation of SOC was not positive (Table 3). Such leveling of C stocks after land use change is common also for other practices aimed at mitigating climate change [35,36]. In addition to the inter-annual variations, this is likely attributed to the low cultivation density, thus a relatively low biomass yield on the M-20 field, and consequently limited residues returning to the soil surface.

### Advocating a systematic evaluation on changes of all relevant soil properties

The potential impacts of Miscanthus on soil characteristics were also reflected by the pH values and nutrient concentrations at the sampling sites. While the pH values in the Miscanthus fields observed in this study were still within the acceptable limits for agricultural soils (Fig 4), they decreased compared with grassland, especially in the topsoil. This suggested that Miscanthus cultivation bears the risk to cause soil acidification, despite the compensation of agricultural lime applied every five years. A similar pattern was observed by Foereid et al. [27] on Miscanthus fields with different ages in Denmark, further highlighting the long-term impacts of Miscanthus on soil sustainability. Zimmermann [37] even detected a negative relationship between pH and SOC concentration on Miscanthus fields. In addition, nutrients, such as P and K, also accumulated in the topsoil (Fig 5). This could be explained by the return of previously fixed elements in residues and stems back to the surface soil after harvesting, and the lack of tillage practices to transfer them further down to deeper layers. Such increased acidity and P and K concentrations in the surface soil may not be of great relevance to the currently growing Miscanthus. However, the changed soil quality may have consequences to crops following the removal of Miscanthus at the end of its life cycle. This is essential to adjusting the regional agriculture management in the Upper Rhine Region, which aims to have flexible land use purposes to ensure the local food supply and confront future climate change [38].

## Conclusion

Our results showed that Miscanthus cultivation could potentially increase the SOC concentration compared to grassland. However, the benefits of SOC sequestration were much more significant in the surface soil (up to 69% more) than in deep layers (only 7% more), which could be attributed to the accumulation of Miscanthus residues in the soil surface. Therefore, our results caution the use of the changes of SOC on the surface soil to estimate the net benefits of Miscanthus cultivation in terms of GHGs emission reduction. In addition, greater SOC sequestration in the upper layers of the Miscanthus fields also meant an increased availability of SOC for decomposition and potentially leading to a new equilibrium over time.

In addition, no matter how the SOC changes, accumulating or depleting, their potential contribution to offset fossil fuel should not be over-accounted for. In particular, the risk of acidification and exceeded contents of P and K adds another precaution to the environmental impacts of Miscanthus cultivation, which is necessary to take into account when adjusting the land use policy in the Upper Rhine Region. While our study was based on regional agro-ecosystems, bearing limitation in upscaling, the changes of soil quality observed in this study highlighted the necessity to systematically evaluate soil sustainability so as to comprehensively understand the environmental impacts of long-term Miscanthus cultivation to both current and future land use changes.

## Supporting information

S1 Table Original data of soil organic carbon concentration, 13C compositions, C:N ratios, soil respiration rates and pH in different layers from the Grassland and Miscanthus field. (XLSX)
